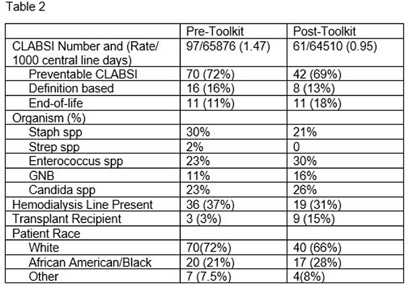# Bundle-Up to Prevent CLABSIs : Analysis of CLABSIs Pre and Post Toolkit Implementation

**DOI:** 10.1017/ash.2025.296

**Published:** 2025-09-24

**Authors:** Chelsea Fauver, Nora Colburn, Justin Smyer, Shandra Day

**Affiliations:** 1 The Ohio State University Wexner Medical Center; 2 The Ohio State University; 3 The Ohio State University Wexner Medical Center; 4 Ohio State University

## Abstract

**Background:** Central line associated bloodstream infections (CLABSIs) are a preventable healthcare-associated infection. Evidence shows implementation of evidence-based bundled infection prevention strategies can reduce CLABSIs. We reviewed the impacts of a CLABSI prevention toolkit on CLABSI rates as well as compliance with key prevention practices. **Methods:** A CLABSI Prevention Bundle Toolkit was implemented in December 2023 at a quaternary care academic medical center. The toolkit delineated the elements of the bundle, including hand hygiene, daily review of line necessity, daily chlorhexidine gluconate (CHG) topical treatment, aseptic technique for insertion and maintenance, along with the responsible party for each task and educational resources for staff and patients. Additionally, the toolkit required weekly audits of CLABSI bundle by individual units and a multidisciplinary meeting to debrief each CLABSI to identify opportunities and successes. Analysis of compliance with key prevention practices, CLABSI rates and clinical details was completed before (December 2022 – November 2023) and after (December 2023 – November 2024) implantation of the toolkit. **Results:** Compliance with key prevention practices pre- and post-toolkit implementation is detailed in Table 1. There was a 37% reduction in CLABSI rate pre- and post-toolkit implementation as shown in Table 2. Clinical details including CLABSI classification as preventable, end-of-life or definition-based (Hsueh, Maurice and Uslan, ICHE 2022), organism, dialysis, transplant status and patient race are detailed in Table 2. **Conclusions:** CLABSI prevention bundles have been shown to reduce CLABSI, but implementation and compliance of the bundle can be challenging. A toolkit which outlines required tasks, responsible parties, regular audits and debriefs after CLABSI can help support healthcare teams in successful implementation of the full CLABSI bundle. Following the bundle toolkit implementation there was improvement in rates of CHG treatment and line necessity review with an overall decrease in CLABSI rates. Not all process measures included in the toolkit are able to be quantified so likely additional factors contributed to the reduction in CLABSI rates. Overall, there did not appear to be a difference in the types of CLABSIs, organisms or patient demographics in the pre and post-toolkit groups although there were more CLABSIs in transplant patients post-toolkit suggesting a complex patient population. A comprehensive toolkit can aide in implementation of a multi-faceted prevention bundle, provide a structure for accountability and help improve patient outcomes.

**Figure:**
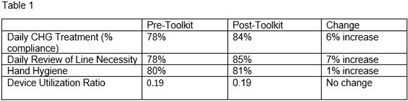

**Figure:**